# Spontaneous empyema and brain abscess in an intensive care population: clinical presentation, microbiology, and factors associated with outcome

**DOI:** 10.1007/s00701-022-05241-7

**Published:** 2022-05-27

**Authors:** Sabeth Dietler, Jan Willms, Giovanna Brandi, Sophie Wang, Astrid Burkerth, Emanuela Keller

**Affiliations:** 1 Neurocritical Care Unit, Institute of Intensive Care Medicine, University Hospital Zurich, Zurich, Switzerland; 2 Neurocritical Care Unit, Dept. of Neurosurgery and Institute of Intensive Care Medicine, University Hospital Zurich, Zurich, Switzerland

**Keywords:** Brain abscess, Spontaneous intracranial empyema, Microbiological findings, Source of infection, Outcome relevant risk factors, Specification of antibiotics

## Abstract

**Background:**

Data on critically ill patients with spontaneous empyema or brain abscess are limited. The aim was to evaluate clinical presentations, factors, and microbiological findings associated with the outcome in patients treated in a Neurocritical Care Unit.

**Methods:**

In this retrospective study, we analyzed 45 out of 101 screened patients with spontaneous epidural or subdural empyema and/or brain abscess treated at a tertiary care center between January 2012 and December 2019. Patients with postoperative infections or spinal abscess were excluded. Medical records were reviewed for baseline characteristics, origin of infection, laboratory and microbiology findings, and treatment characteristics. The outcome was determined using the Glasgow outcome scale extended (GOSE).

**Results:**

Favorable outcome (GOSE 5–8) was achieved in 38 of 45 patients (84%). Four patients died (9%), three remained severely disabled (7%). Unfavorable outcome was associated with a decreased level of consciousness at admission (Glasgow coma scale < 9) (43% versus 3%; *p* = 0.009), need of vasopressors (71% versus 11%; *p* = 0.002), sepsis (43% versus 8%; *p* = 0.013), higher age (65.1 ± 15.7 versus 46.9 ± 17.5 years; *p* = 0.014), shorter time between symptoms onset and ICU admission (5 ± 2.4 days versus 11.6 ± 16.8 days; *p* = 0.013), and higher median C-reactive protein (CRP) serum levels (206 mg/l, range 15–259 mg/l versus 17.5 mg/l, range 3.3–72.7 mg/l; *p* = 0.036). With antibiotics adapted according to culture sensitivities in the first 2 weeks, neuroimaging revealed a progression of empyema or abscess in 45% of the cases.

**Conclusion:**

Favorable outcome can be achieved in a considerable proportion of an intensive care population with spontaneous empyema or brain abscess. Sepsis and more frequent need for vasopressors, associated with unfavorable outcome, indicate a fulminant course of a not only cerebral but systemic infection. Change of antibiotic therapy according to microbiological findings in the first 2 weeks should be exercised with great caution.

## Introduction

In developed countries, severe intracranial infections such as epidural or subdural empyema and pyogenic brain abscess occur with an incidence of 0.4 to 0.9 per 100,000 inhabitants and per year [5]. Despite diagnostic and therapeutic advances, the case fatality rate remains high with an estimated mortality rate of 5–15% [3, 6, 12], as well as long-term risk of death remains increased in patients with brain abscess [2]. So far, only one study analyzing a patient series from the 1990s focused on an intensive care population with most severe intracranial infections [19].

The purpose of the present study is to determine clinical features, complications, and factors associated with the outcome in critically ill patients with spontaneous empyema or brain abscess treated in a Neurocritical Care Unit.

## Methods

Medical records of patients with radiologically, microbiologically, or intraoperatively verified epidural and/or subdural empyema or pyogenic brain abscess admitted to the Neurocritical Care Unit, University Hospital of Zurich between January 2012 and December 2019 were retrospectively reviewed. Patient records were assessed for various variables such as baseline characteristics, comorbidities, origin of infection, radiological and laboratory findings including microbiology, as well as for treatment characteristics, e.g., neurosurgical interventions and antibiotics. The outcome was determined using the Glasgow outcome scale extended (GOSE), assessed at follow-up visits in the outpatient clinic [14, 20]. Sepsis was defined as life-threatening organ dysfunction according to International Consensus Definition [17].

This retrospective study was approved by the local ethics committee. Since a significant number of patients have deceased, the requirement for informed consent was waived.

For statistical analysis, the outcome was dichotomized into favorable (GOSE 5–8) and unfavorable outcome (GOSE 1–4) [14, 23]. Where applicable, data are presented as median with interquartile range (IQR) or mean with 95% confidence interval (CI). Differences between groups were compared using the Fisher’s exact, Mann–Whitney *U* or *t*-test where appropriate. Statistical analysis was performed using SPSS statistics 26.0 software (IBM Corporation, Armonk, New York, USA).

## Results

### Patient characteristics

Medical records from 101 patients were reviewed. Patients with postoperative infections (*n* = 43), spinal abscess (*n* = 9), and/or declined general consent (*n* = 4) were excluded, leaving a cohort of 45 patients for analysis.

Patient characteristics are given in Table 1. Most patients were male (60%) and the mean age was 49.7 years (± 18.3). Twenty-one patients (47%) suffered from relevant comorbidities, such as alcohol or drug abuse (16%) or previous infectious disease (29%), one patient each with Hep B, Hep C, HIV, otitis media, aspergilloma of the lung, acute urinary tract infection, *Pneumococcus *meningoencephalitis, a dental abscess, and one patient with an undefined infection. Three patients were treated with immunosuppressants; one after bilateral lung transplantation due to cystic fibrosis, the others were treated with steroids for sarcoidosis and trigeminal neuralgia. An important reason for Intensive Care Unit (ICU) admission was imminent brain herniation or midline shift (*n* = 22, 49%), seizures (*n* = 8, 19%), and a reduced level of consciousness (Glasgow coma scale (GCS) < 9, *n* = 4; 9%). Eleven patients (27%) presented with the classical triad of fever, headache, and focal neurologic deficits. In 16 patients (35%), values for C-reactive protein (CRP), procalcitonin (PCT), and leucocyte count were in a normal range.

**Table 1:** Characteristics of patients with spontaneous empyema or abscess

	All patients (*n* = 45)	Patients with favorable outcome (*n* = 38)	Patients with unfavorable outcome (*n* = 7)	*p*-value
Age in years; mean (SD)	49.7 (18.3)	46.9 (17.5)	65.1 (15.7)	0.014
Male; *n* (%)	27 (60%)	24 (63%)	3 (43%)	0.415
Comorbidities; *n* (%)	21 (46.7%)	16 (42%)	5 (71%)	0.158
History of infectious disease^A^	9 (29%)	7 (18%)	2 (29%)	0.605
Cancer	5 (11.1%)	3 (8%)	2 (29%)	0.114
Alcohol/drug abuse	6 (16.2%)	4 (13%)	2 (40%)	0.126
Diabetes	3 (6.7%)	2 (5%)	1 (14%)	0.385
Immunosuppressive therapy	3 (6.7%)	2 (5%)	1 (14%)	0.385
Heart disease	2 (4.4%)	2 (5%)	0	0.539
Hereditary hemorrhagic telangiectasia	1 (2.2%)	1 (3%)	0	0.668
Symptoms before hospital admission; *n* (%)				
Symptoms duration in days; mean (SD)	10.8 (15.9)	11.6 (16.8)	5.0 (2.4)	0.013
Headache	31 (74%)	28 (76%)	3 (60%)	0.460
Fever	21 (51%)	17 (49%)	4 (67%)	0.418
Altered mental state	20 (44%)	15 (40%)	5 (71%)	0.122
Focal neurological deficit	34 (76%)	28 (74%)	6 (86%)	0.501
Triad (fever, headache, FND)	11 (27%)	8 (23%)	3 (50%)	0.171
Reason for ICU admission; *n* (%)				
Mechanical ventilation due to deterioration	4 (9%)	2 (5%)	2 (29%)	0.049
Decreased level of consciousness (GCS < 9)	4 (9%)	1 (3%)	3 (43%)	0.009
Seizure	8 (19%)	7 (18%)	1 (20%)	0.954
Midline shift or signs of imminent brain herniation	22 (49%)	18 (47%)	4 (57%)	0.638
Laboratory values; median (IQR)				
CRP in mg/l; median (IQR)	19 (4.5–99.00)	17.5 (3.35–72.75)	206 (15–259.00)	0.036
PCT in ug/l; median (IQR)	0.57 (0.12–0.56)	3.22 (0.11–4.45)	0.88 (0.1025–5.78)	0.889
Leucocyte count G/l; median (IQR)	9.76 (7.44–13.68)	9.39 (7.22–12.2)	12.09 (8.40–27.39)	0.223

*CRP*, C-reactive protein; *FND*, focal neurological deficit; *ICU*, intensive care unit; *IQR*, interquartile range; *GCS*, Glasgow coma scale; *PCT*, procalcitonin; *SD*, standard deviation

^A^One case each with acute urinary tract infection, pulmonary aspergilloma, hepatitis B and C, HIV, and tuberculosis

The outcome was assessed on average after 217 days (± 133) after hospital discharge, while five patients were not available for a follow-up longer than 3 months. Of the 45 patients, 38 (84%) survived with a favorable outcome. In seven patients (16%), we recorded an unfavorable outcome (GOSE 1 to 4), four of them died (9%) and three patients (67%) survived severely disabled (GOSE 4). No patient survived with GOSE 2 or 3.

Patients with unfavorable outcome were older (65.1 ± 15.7 years; *p* = 0.014) and time between symptoms onset and ICU admission (5 ± 2.4 days; *p* = 0.013) was shorter. The other factors included a decreased level of consciousness defined as GCS < 9 at admission (43% versus 3%; *p* = 0.009) and higher frequency of mechanical ventilation (29% versus 5%; *p* = 0.049).

### Source of infection

Characteristics of the infectious origin are given in Table 2. In 27 patients (60%), the origin of the infection could be identified, which was focal in 49% of the cases, with sinusitis being the most frequent source (24%). In patients with unfavorable outcome, the source of infection tended to be identifiable more frequently (six among seven patients; 86%) compared to those with favorable outcome (21 among 38 patients; 55%) but without statistical significance. Due to the small numbers, no statistical comparisons were made in the subgroups.

**Table 2:** Origin of infection

	All patients (*n* = 45)
Origin identified	27 (60%)
Focal origin	22 (49%)
Sinusitis	11 (24%)
Mastoiditis/otitis	6 (13%)
Dental	5 (11%)
Systemic origin	5 (11%)
Bacterial endocarditis	2 (4%)
Pulmonary empyema/abcess	1 (2%)
Others (urosepsis/liver abscess)	2 (4%)

### Abscess localization

The main localizations of empyema and abscesses are given in Table 3. Their expansion could involve several lobes. In 69%, the empyema or abscess was right-sided and in 9% bilaterally, most often in frontal and temporal lobes. Multifocal abscesses were present in 20%. Concomitant cerebral sinus thrombosis occurred in three cases, two of them had an unfavorable outcome.

**Table 3:** Localization of empyema or abscess

	All patients (*n* = 45)
Left-sided	10 (22%)
Right-sided	31 (69%)
Bilateral	4 (9%)
Empyema	17 (38%)
Epidural	5
Subdural	12
Single abscess	34 (76%)
Frontal	14
Temporal	14
Parietal	13
Occipital	2
Cerebellar	0
Brainstem	3
Multifocal abscesses	9 (20%)
Frontal	5
Temporal	2
Parietal	4
Occipital	1
Cerebellar	1
Brainstem	0

### Microbiology

Detailed microbiological results are listed in Table 4. Antibiotic therapy was already initiated in 21 patients (47%) before ICU admission. Therefore, in almost half of the cases, microbiological diagnostics from the abscess material, cerebrospinal fluid (CSF), or blood cultures were performed while the patient was under ongoing antibiotic therapy. Cultures from the abscess material were obtained in 44 of 45 cases and were positive for microorganisms in 38 of 44 cases (86%). In one patient with bacterial meningoencephalitis and secondary abscess and positive CSF findings, no additional abscess puncture was performed. In 15 patients (75%), bacterial growth was detected in the abscess despite prior antibiotic therapy (*n* = 20). Of the patients without prior antibiotic therapy (*n* = 24), bacteria could be found in 23, i.e., 95% of the cases. In 18 patients (41%), multiple microorganisms were detected and mostly gram-positive bacteria were found (59%). Anaerobes were mostly cultivated in mixed infectious disease (13 cases, 30%). One patient, without any immunodeficiency, was diagnosed with aspergillus abscess. In a patient with bilateral lung transplanted patient, *Achromobacter xylosoxidans* and—as an opportunistic pathogen—*Lichtheimia corymbifera* were detected. Blood cultures were positive in 14 of 32 patients (44%).

**Table 4:** Microbiology of abscess material and blood cultures

	Abscess material (*n* = 44)		Blood cultures (*n* = 32)
Antibiotic therapy before admission	21 (47.7%)		15 (46.8%)
Negative cultures	7 (15.9%)		18 (56.3%)
Single microorganism	20 (46%)		10 (31%)
Multiple microorganism	18 (41%)		4 (13%)
Gram-positive microorganisms^A^	26 (59%)		15 (50%)
* Strep. anginosus* group	22 (50%)		6 (19%)
* Strep. pneumoniae*	0		1 (3%)
* Strep. pyogenes*	2 (5%)		2 (6%)
Coag-neg. *Staph.* sp.	4 (9%)		3 (9%)
* Klebsiella pneumoniae*	1 (2%)		0
* Staph aureus*	1 (2%)		2 (6%)
Other gram-positive bacteria	3 (7%)		1 (3%)
Gram-negative bacteria^A^	11 (25%)		1 (3%)
* Camphylobacter* sp.	0		0
* E. coli*	1 (2%)		1 (3%)
Proteus sp.	0		0
HACEK-Group	9 (21%)		0
Other gram-negative bacteria	1 (2%)		0
Anaerobes^A^	13 (30%)		2 (6%)
Bacteroides sp.	1 (2%)		0
* Fusobacterium* sp.	5 (11%)		1 (3.1%)
* Prevotella* sp.	1 (2%)		0
* Proprionibacterium* sp.	0		0
Other anaerobes	7 (16%)		1 (3.1%)
Fungi	2 (5%)		**0**
* Aspergillus* sp.	1 (2%)		0
* Candida* sp.	0		0
* Cryptococcus neoformans*	0		0
Other fungi	1 (2%)		0

^A^The sum does not correspond to the number of species, as multiple occurrences of the same bacterial species in one culture was only counted once

Of these, only four cultures with evidence of a single bacterial strand in the abscess and blood cultures were the same. In three cases with polybacterial detection, only partially identical bacteria were found in the abscess and in the blood cultures. In four of the 12 patients, in whom CSF was analyzed (in nine times via lumbar puncture, in three times via external ventricular drain) bacterial growth was proven, but only in one the same bacteria were detected as in the abscess.

### Characteristics of treatment and complications

One patient with small abscesses underwent only conservative treatment with long-term antibiotics, another was abscess punctured in an external hospital. The others had at least one neurosurgical intervention with empyema evacuation and/or microsurgical abscess aspiration. A source control surgical procedure (as mastoidectomy, sinus drain, dental extraction) was performed in 22 cases. Due to progression, neurosurgical revisions had to be performed in nine patients (21%), and in another seven (18%) even three or more re-interventions had to be performed. External ventricular drain due to hydrocephalus was inserted in nine patients (21%), while no patient needed a permanent ventriculoperitoneal shunt.

Initial antibiotic therapy usually consisted of ceftriaxone combined with metronidazole intravenously. Change to oral antibiotic therapy depended on clinical condition. Intravenous and oral antibiotic treatment in total was performed for at least 6 weeks.

**Fig. 1:**
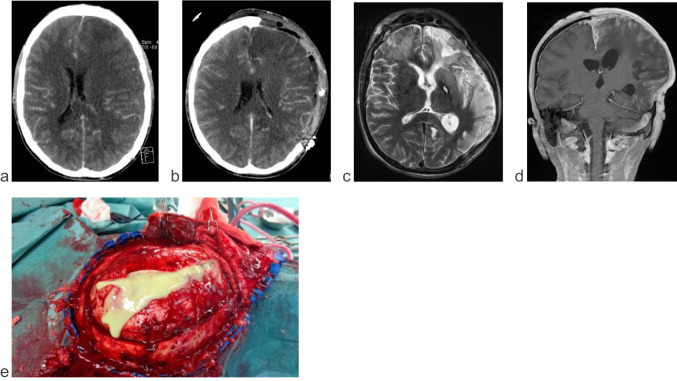
Patient with intracranial space-occupying left hemispheric and interhemispheric subdural empyema**. a** Contrast-enhanced axial scan CT at admission. **b** Follow-up CT scan 7 days after emergency hemicraniectomy and evacuation of left hemispheric empyema. **c** T2-weighted axial scan MRT and **d** T2-weighted FLAIR coronar scan, 4 weeks after admission. **e** Intraoperative situs with emission of pus after opening of the dura

Complications during the ICU stay are given in Table 5. In 20 patients, the antibiotic treatment was adapted to culture sensitivities in the first 2 weeks. In nine of these cases (45%), CT- or MR-imaging established a renewed deterioration and progression of empyema or abscess, and antibiosis had to be broadened again. This course of disease was relatively more common in patients with poor outcome (in 57% of cases). Elevated intracranial pressure (ICP) occurred in nine patients but could be controlled in all patients with osmotherapy and, finally, decompressive surgery in four patients (Fig. 1). Due to the space-occupying effect of the brain abscess with midline shift, 26 patients (58%) were treated with steroids in the early phase.

**Table 5:** Complications during ICU stay

	All patients (*n* = 45)	Patients with favorable outcome (*n* = 38)	Patients with unfavorable outcome (*n* = 7)	*p*-value
Need for treatment of elevated ICP	9 (20%)	6 (16%)	3 (43%)	0.104
Seizure	8 (18%)	5 (13%)	3 (43%)	0.062
Hydrocephalus	4 (9%)	3 (8%)	1 (14%)	0.589
Sepsis	6 (13%)	3 (8%)	3 (43%)	0.013
Renal failure	2 (4%)	1 (3%)	1 (14%)	0.174
Vasopressor support	9 (20%)	4 (11%)	5 (71%)	0.002
Thromboembolic complications	4 (9%)	4 (11%)	0	0.374
Nosocomial pneumonia	3 (7%)	2 (5%)	1 (14%)	0.385
Progression of empyema or abscess after adaption of antibiotic therapy according to culture sensitivities	9 (20%)	5 (13%)	4 (57%)	0.005

*ICP*, intracranial pressure

Based on electroencephalography (EEG) findings or after seizures, 24 patients (53%) were treated with antiepileptics during the ICU stay. Among them were two patients with previously known epilepsy. Six and seven patients respectively had at least one epileptic seizure on admission (one patient was postulated to have a seizure, but the therapy was stopped in the course of treatment due to improbable epilepsy). In addition, seven patients had epileptic seizures during the course of therapy. In nine patients, prophylactic antiepileptic therapy was started despite the absence of clinical seizures, and ETPs were detected in only two of these. No status epilepticus occurred. In the long-term follow-up, 40% of the patients remained under antiepileptic therapy for at least 6 months. Nine patients had to be supported by vasopressors and six developed sepsis, both occurred more frequently in the group with unfavorable outcome (71% and 43% respectively).

## Discussion

In this specific, intensive care population with spontaneous empyema or brain abscess, the case fatality rate of 9% was in line with the range in general patient population [4, 7]. Three patients survived severely disabled, leading to an unfavorable outcome in 16%.

Unfavorable outcome was associated with higher age, the presence of comorbidities and decreased level of consciousness at admission. Lange et al. found, by conducting a retrospective study of 47 patients with primary brain abscesses, a significantly worse outcome at an age over 60 years [11]. Rapid neurological deterioration may be a highly important predictor of clinical outcome [9, 10, 13, 15, 16]. In a retrospective study of 51 patients, Ko et al. found in multivariate analyses a low initial GCS score < 13 to be independently associated with unfavorable outcome at hospital discharge [10]. In a series of 113 patients by Widdrington et al., including cases with recent surgery or trauma, reduced GCS, focal neurological deficit, and seizures at presentation were independently associated with an unfavorable clinical outcome [24]. Also in accordance with the literature, we found the mean time between occurrence of first symptoms and hospitalization to be significantly shorter in those with poorer outcomes [9, 16]. Higher CRP serum levels in patients with unfavorable outcome indicate a fulminant course of the infection. Accordingly in patients with unfavorable outcome vasopressors were administered more frequently and, similar to the findings of Tseng et al., sepsis occurred more often [22].

Our data indicate that the source of infection may be difficult to be identified. Even the diagnosis of spontaneous empyema or brain abscess can be difficult. The typical triad of fever, headache, and focal neurological deficits in our patient population was only present in about a fourth of the cases and 35% of the patients had normal values for CRP, PCT, and leucocyte count. These results are in accordance with the findings of a meta-analysis by Brouwer et al., covering 9699 patients in 123 studies which found the classical triad to be present in only 20% of the cases [4]. Therefore, as also suggested by other authors, systemic laboratory parameters such as leucocyte count, CRP, and PCT are not reliable indicators of intracranial empyema or brain abscess [4, 8, 10].

At ICU admission, microbiological diagnostics can also be impaired. Almost half of our patients were already under antibiotic therapy and the sensitivity of blood cultures is low. Even if lumbar puncture is possible with respect to imminent brain herniation, CSF diagnostics rarely tend to be sensitive to identify the microorganisms. Empyema and abscess evacuation must be pursued as quickly as possible also to adapt the empirically started antibiotic therapy. In our study, cultures from the abscess material were positive for microorganisms in more than 80% of the patients. In about 40% multiple microorganisms were detected in the abscess material with anaerobes being the most frequent microorganisms. Rare opportunistic pathogens might be identified in patients under immunosuppressive therapy. The immediate search for the origin of infection includes imaging of the sinuses and the mastoid by means of computed tomography (CT) or magnetic resonance imaging (MRI). High-resolution CT will allow to identify odontogenic foci as potential source of infection. Positive blood cultures and long-term systemic signs of infection require repeated transesophageal echocardiography.

As in other studies, *Streptococcus anginosus* was the microorganism most frequently identified in the abscess material [6, 7, 18, 24]. Multiple microorganisms, however, were present in 40%. Therefore, adaption of antibiotic therapy according to culture sensitivities in the first 2 weeks should be exercised with great caution. Almost one half (45%) of the patients in our cohort in whom the therapy was changed according to the microbiological results showed a new progression of empyema or abscess requiring a renewed escalation of antibiotics or surgical revision. In recent reviews, it is suggested that change of antibiotics based on microbiological findings is only safe if samples are not obtained in patients under antibiotic pretreatment and are processed optimally or when primary diagnosis is endocarditis [6, 18]. Further microbiological evaluations may benefit using metagenomic analysis, as extended metagenomic evaluations (polymerase chain reaction (PCR) and 16S ribosomal DNA amplification) from brain abscess specimens can detect uncultured, especially slow-growing and fastidious bacteria [1, 21]. Further studies, however, also have to investigate the clinical significance of pathogens identified only through metagenomic analysis but not by conventional cultures [18].

This study has several limitations. In addition to the study design of a single-center, retrospective, observational study, it has a small sample size with 45 patients and only seven patients had an unfavorable outcome prohibiting multivariate statistics. The small number of five patients with underlying systemic source of infection might be underestimated, since only patients in a Neurocritical Care Unit were analyzed. In other studies, hematogenic spread is postulated to occur in 15% to 25% [3, 24]. Patients primarily suffering from endocarditis, pulmonary, or abdominal infection, and small cerebral abscesses might have been treated in other ICUs. Outcome data were collected based on reports of consultations at the outpatient clinic. A standardized, precisely timed follow-up with the assessment of the outcome scores would provide more accurate and precise information about the patients’ conditions and should be a key element in a possible prospective examination.

In conclusion, in this specific population of critically ill patients with spontaneous empyema or brain abscess, unfavorable outcome was associated with higher age, decreased level of consciousness upon admission, as well as shorter time between symptoms onset and ICU admission. Higher inflammatory markers in serum, more frequent need for vasopressors, and sepsis, associated with unfavorable outcome, indicate a fulminant course of a not only cerebral but systemic infection.
